# Beneficial effects of daytime high-intensity light exposure on daily rhythms, metabolic state and affect

**DOI:** 10.1038/s41598-020-76636-8

**Published:** 2020-11-13

**Authors:** Carmel Bilu, Haim Einat, Paul Zimmet, Vicktoria Vishnevskia-Dai, Noga Kronfeld-Schor

**Affiliations:** 1 School of Zoology, Tel-Aviv University, Tel Aviv, Ramat Aviv, Israel; 2 School of Behavioral Sciences, Tel Aviv-Yaffo Academic College, Tel-Aviv, Israel; 3 Department of Medicine, Monash University, Melbourne, VIC Australia; 4 Ocular Oncology and Autoimmune Service, The Goldschleger Eye Institute, The Chaim Sheba Medical Center, Tel-Hashomer, Israel; 5 Sackler Faculty of Medicine, Tel-Aviv University, Tel-Aviv, Israel

**Keywords:** Type 2 diabetes, Circadian regulation

## Abstract

While the importance of the circadian system to health and well-being is extensively studied, the role of daylight exposure in these interactions is relatively poorly understood. Here we show, using a diurnal animal model naturally exposed to daylight, that daily morning exposure to 3000 lux, full spectrum electric light has beneficial health effects. Compared with controls, sand rats (*Psammomys obesus*) subjected to morning light treatment demonstrate daily rhythms with high peak to trough difference in activity, blood glucose levels and per2 gene expression in the suprachiasmatic nucleus, pre-frontal cortex, kidney and liver. The treated animals were also healthier, being normoglycemic, having higher glucose tolerance, lower body and heart weight and lower anxiety- and depression-like behavior. Our results suggest that exposure to high intensity light is important for the proper function of the circadian system and well-being, and are important in face of human's low exposure to daylight and extensive use of artificial light at night.

## Introduction

Exposure to bright light in the morning (bright light treatment or therapy, BLT) is a well-established treatment for mood disorders such as seasonal affective disorder (SAD), major depressive episodes from unipolar or bipolar disorders, and sleep disorders including shift work and sleep phase disorders^1–10^. Despite the use of this effective treatment for decades, the underlying biological mechanism of its beneficial effect remains unclear. It is assumed to act through intrinsically photosensitive retinal ganglion cells (ipRGCs) containing melanopsin^11–13^. These photoreceptors transfer information to non-visual nuclei in the brain, including areas that have been associated with mood and anxiety and the suprachiasmatic nuclei (SCN), the central biological clock, affecting mood both directly and through the circadian system^7,11–16^.

The circadian system is a major regulator of almost every aspect of the body function, including metabolism. Studies in humans and animal models suggest close links between circadian dysfunction and pathological conditions such as the metabolic syndrome or the circadian syndrome (a recently suggested term encompassing the key components of the metabolic syndrome and its main comorbidities: sleep disturbances, depression, steatohepatitis and cognitive dysfunction^17–19^), including obesity and T2DM^18,20–25^. Based on the observed effects of BLT on affective disorders and the hypothesis that its beneficial effect acts, at least partially, through the circadian system, a few studies tested the effect of BLT on mood and insulin sensitivity in patients with T2DM and depression^26^. These study concluded that BLT may be a promising treatment for a subgroup of highly insulin resistant individuals with T2DM. Moreover, two separate case reports found that BLT for patients with T2DM and SAD ameliorated the SAD symptoms, and increased insulin sensitivity^27,28^. However, a study comparing healthy and obese men with T2DM found that in healthy men BLT did not affect fasting or postprandial plasma glucose levels but resulted in an increase in fasting and postprandial plasma triglyceride levels. In men with T2DM, it increased fasting and postprandial plasma glucose, had no effect on fasting plasma triglycerides, but increased postprandial plasma triglyceride levels^29^. Together, these results suggest that BLT has direct metabolic effects, possibly by affecting the circadian system, and warrants further studies in order to better describe the response to BLT and its mode of action.

During the last years, we have been using sand rats (*Psammomys obesus*) as a diurnal model for studying the relations between circadian rhythms, depressive- and anxiety-like behavior, T2DM, cataracts, cardiac hypertrophy and cardio-metabolic risk factors, which we described as "the circadian syndrome"^17–19,30–33^. In the laboratory, when sand rats are fed standard rodent food, they develop T2DM characterized by enhanced insulin secretion and insulin resistance at the early stages and insulin deficiency at an advanced stage^34,35^. Under these conditions, they also show a small peak-trough difference (PTD) in rhythmicity of activity, physiology and gene expression, and display depressive- and anxiety-like behaviors^17,33,36^. Moreover, keeping sand rats under short photoperiod (5:19 LD cycle) accelerated the development of these phenotypic features compared to neutral photoperiod (12:12 LD cycle)^33^, including adipocyte hypertrophy and dysfunction coupled with a pro-inflammatory milieu^19^. In contrast, sand rats kept in laboratory cages outdoors where they are exposed to natural environmental conditions, many of which have daily fluctuations, they are diurnally active, exhibit robust daily rhythms, and do not develop T2DM or depression-like like behavior^33^. We also found significant correlations at the individual level between anxiety-like behavior and glucose tolerance, and between heart/total body weight ratio and glucose tolerance^17^. Altogether, our results suggest that the observed comorbidity is linked to disturbance in circadian rhythms.

We have previously found that as in humans^37,38^, three weeks of morning, full spectrum electrical BLT ameliorated the depressive- and anxiety-like behavior caused by keeping sand rats under short photoperiod (5:19 light:dark lighting regimen), and that morning BLT had a significantly stronger effect compared with evening BLT^32,39,40^. We now present a new study in which we hypothesized that long term 1 h of morning BLT will prevent the development of the circadian syndrome. Our hypothesis is based on four hypotheses and previous findings: First, the hypothesis that BLT prevents circadian rhythm-related disorders (depressive- and anxiety-like behavior) by resynchronizing disrupted circadian rhythms. Second, our findings that BLT ameliorates the depressive- and anxiety-like behavior caused by keeping the sand rats under short photoperiod. Third, our hypothesis that the comorbidity between T2DM, cardiac hypertrophy, depression-and anxiety-like behavior, that we have found in sand rats, is linked to disturbance in circadian rhythms, and fourth, that our finding that when kept outdoors sand rats show robust daily rhythms and do not develop the circadian syndrome may result from the exposure to high intensity, full spectrum natural light during the morning.

## Results

### Activity rhythms

**Figure 1:**
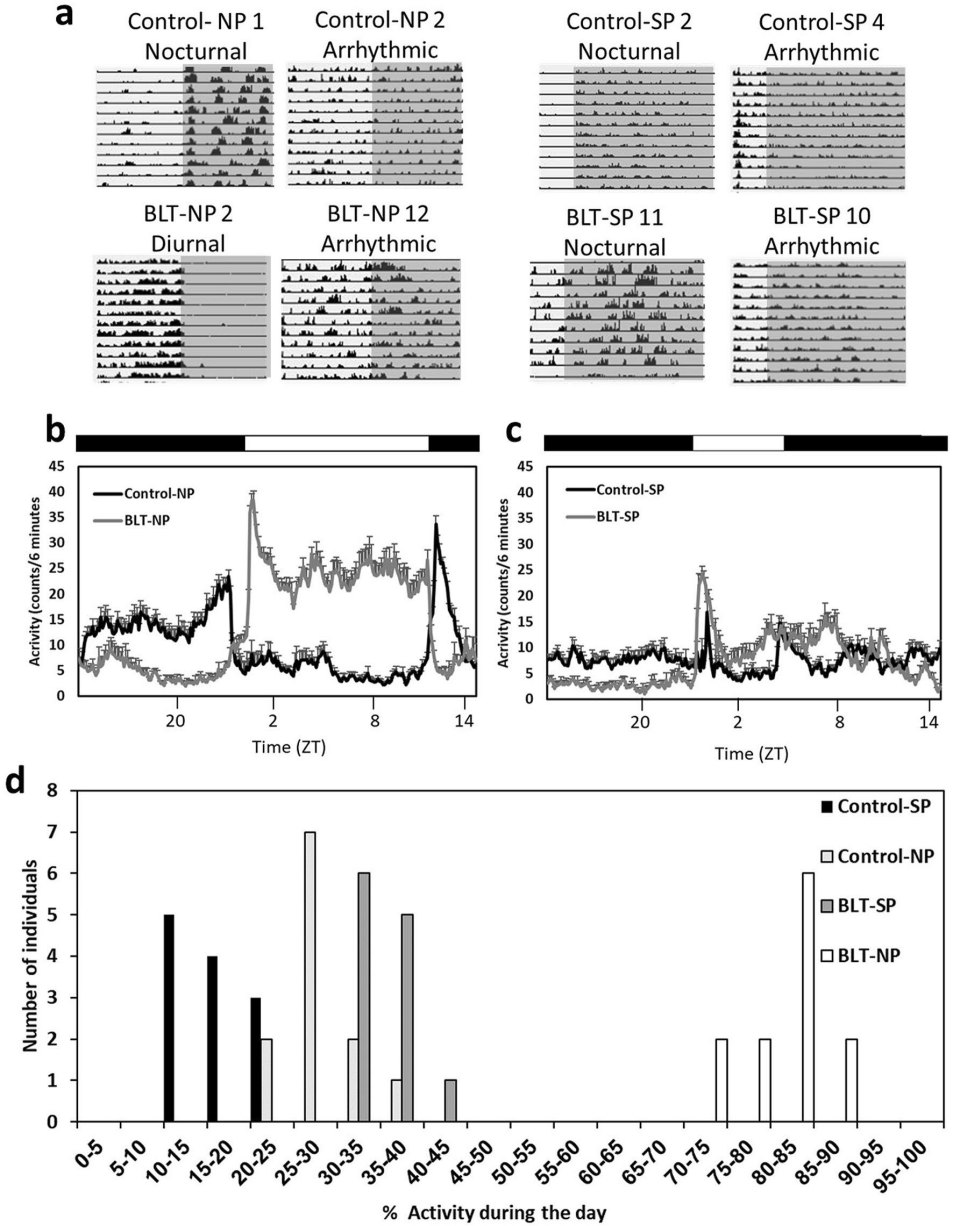
Activity patterns of the different treatment groups during the experiment. (**a**) Representative actograms of two sand rats from each experimental group: neutral photoperiod (Control-NP 1—nocturnal, 2—arrhythmic), neutral photoperiod with BLT (BLT-NP 2—diurnal, 12—arrhythmic), short photoperiod (Control-SP 2—nocturnal,4—arrhythmic), short photoperiod with BLT (BLT-SP 11—nocturnal, 10—arrhythmic). Each row represents one day, depicted one below the other. Dark background represents dark hours. (**b**,**c**) Average daily activity rhythm of control and BLT neutral (**b**) and short (**c**) photoperiod-acclimated sand rats during the last two weeks of the experiment (weeks 16–17). n = 12 per group, Mean ± SEM. The black bar above the figure represents dark hours, the white bar—light hours. (**d**): distribution of % activity during the day relative to 24 h, in the four experimental groups. n = 24.

Control animals kept under NP (neutral photoperiod, 12:12 light:dark) or SP (short photoperiod, 5:19 light:dark), and BLT animals kept under SP were arrhythmic (no significant rhythm detected in a χ^2^ test, see methods) or nocturnal (more than 50% of their activity occurred during the dark phase) (Control-NP (NP, no BLT): 8/11 nocturnal, 3/11 arrhythmic, Control-SP (SP, no BLT): 1/11 nocturnal, 10/11 arrhythmic, BLT-SP (SP with BLT): 4/6 nocturnal, 2/6 arrhythmic) whereas animals kept under NP with BLT were mostly diurnal (more than 50% of their activity occurred during the light phase) [BLT-NP (NP with BLT): 11/12 diurnal, 1/12 arrhythmic]. The Control-SP group had a significantly higher number of arrhythmic animals (compared with BLT-SP χ^2^(1) = 6.2, *p* = 0.01; compared with Control-NP χ^2^(1) = 9.2, *p* = 0.002; Fig. 1).

### Glucose tolerance

**Figure 2:**
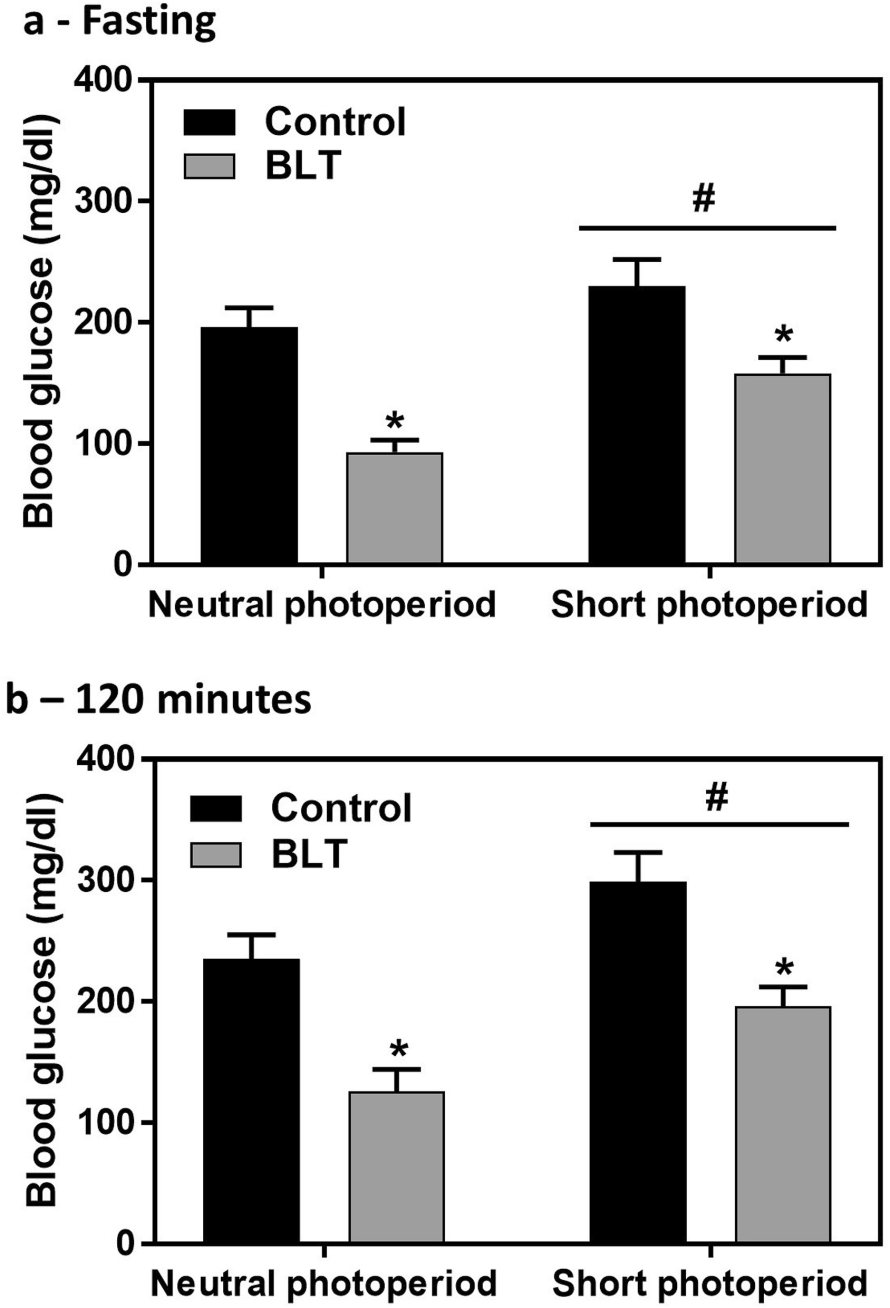
Fasting blood glucose levels (mg/dL) at baseline (**a**) and 120-min post-glucose administration in the oral glucose tolerance test, performed at ZT2, on week 16 (**b**): short photoperiods resulted in increased blood glucose levels, whereas bright light exposure resulted in reduction in glucose levels. n = 31–34 per group, bars represent the Mean ± SEM. ^#^Difference between short and neutral photoperiod, *p* < 0.01. *Difference within each photoperiod, *p* < 0.001.

Data did not follow normal distribution and were analyzed using non-parametric Kruskal–Wallis test. Blood glucose levels were significantly influenced by both photoperiod length and bright light treatment at both baseline (fasting) and 120 min after glucose administration in the oral glucose tolerance test. Short photoperiods resulted in higher fasting glucose levels [H(1,108) = 7.9, *p* = 0.005; Cohen’s d = Cohen’s d = 0.53] whereas bright light exposure resulted in reduction in glucose levels [H(1,108) = 20.0, *p* < 0.001; Conen’s d = 0.94] Fig. 2a;. Similar effects were demonstrated 120 min after the administration of glucose with higher levels in animals maintained at SP [H(1,108) = 7.5, *p* = 0.006; Cohen’s d = 0.51] and lower levels in animals that were exposed to bright light [H(1,108) = 21.5, *p* < 0.001; Cohen’s d = 0.98] (Fig. 2b). Additional data are presented in the supplementary information (Table A1).

### Blood glucose rhythm

**Figure 3:**
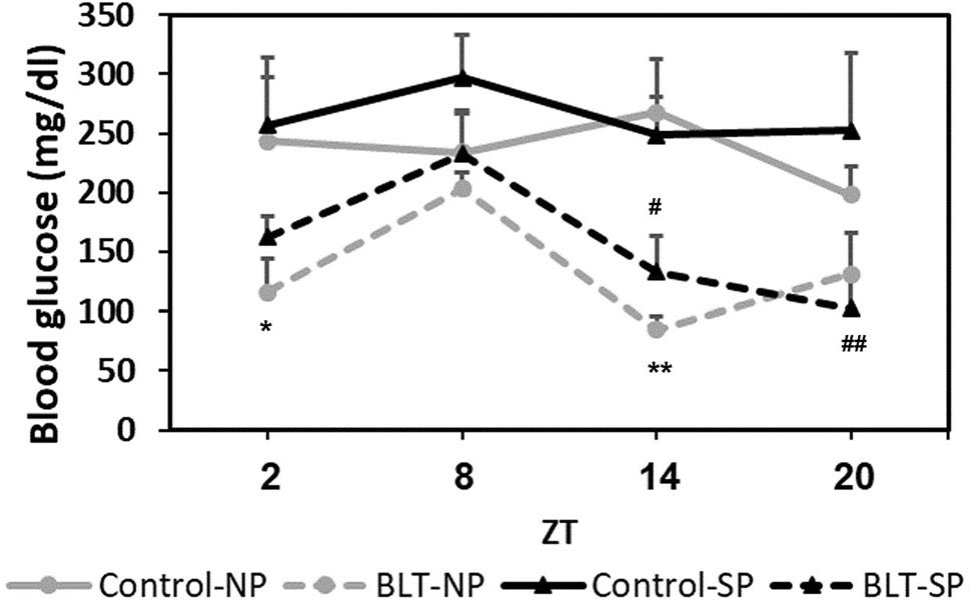
Twenty-four-hour glucose rhythm: Non-fasting blood glucose levels (Mean ± SEM) of animals receiving BLT showed a significant daily rhythm: For BLT-NP glucose levels at ZT8 significantly differ from glucose levels at ZT 2 (*p = 0.03) and at ZT14 (**p = 0.03). For BLT-SP glucose levels at ZT8 significantly differ from glucose levels at ZT 14 (^#^*p* = 0.02) and at ZT20 (^##^*p* = 0.003). Blood glucose levels of sand rats that did not receive BLT showed no significant differences among the four ZTs.

Animals that did not receive BLT did not show a significant daily glucose rhythm, regardless of photoperiod conditions. Animals receiving BLT showed a significant twenty-four-hour blood glucose rhythm under both photoperiods (Statistics: Table 1, Fig. 3). Additional data are presented in the supplementary information (Table A2).

**Table 1:** Statistical results of the effect of photoperiod and BLT on blood glucose levels at different ZTs.

Group	ANOVA	Post-hoc LSD
Control-NP	F(3,29) = 0.56, *p* = 0.65	–
BLT-NP	F(3,24) = 2,65, *p* = 0.07	**ZT2 ≠ ZT8 (p = 0.03)** **ZT8 ≠ 14 (p = 0.015)**
Control-SP	F(3,21) = 0.25, *p* = 0.86	–
BLT-SP	F(3,26) = 3.94, *p* = 0.019	**ZT8 ≠ ZT14 (p = 0.02)** **ZT8 ≠ ZT20 (p = 0.003)** **ZT8 compared with ZT2 (p = 0.051)**

### Activity rhythm and blood glucose levels

**Figure 4:**
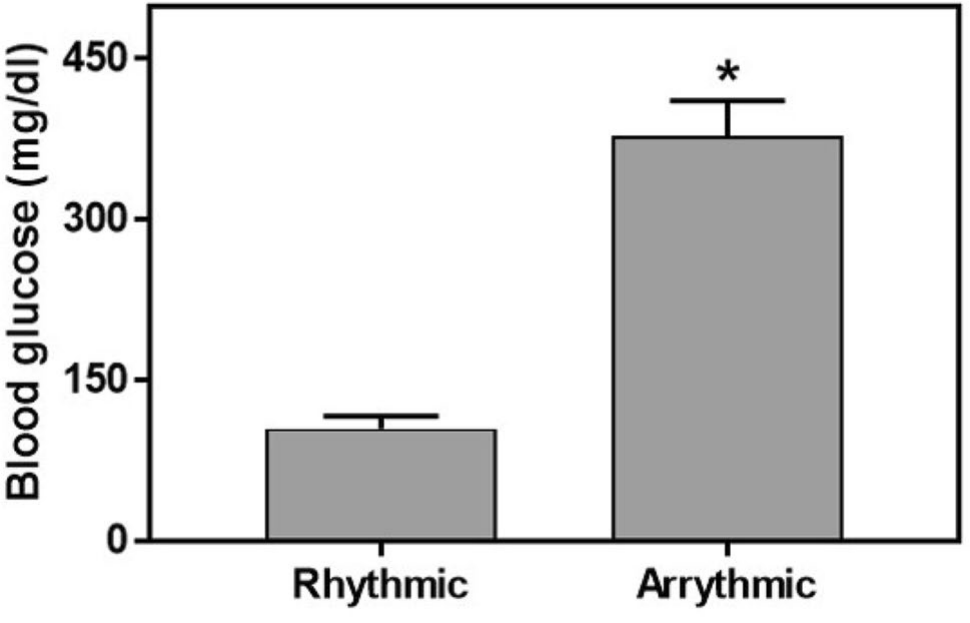
Blood glucose levels (mg/dl, Mean ± SEM) of rhythmic compared with arrhythmic sand rats. *p < 0.0001.

Data did not follow normal distribution and were analyzed using non-parametric Kruskal–Wallis test. There was a significant difference across groups between rhythmic and arrhythmic animals (see methods) in their glucose levels 120 min past glucose administration in the OGTT (H(1,40) = 26.4, *p* < 0.001; Cohen’s d = 2.8) (Fig. 4). This difference was maintained when analysis was performed separately for each group (data not shown). Additional data are presented in the supplementary information (Table A3).

### Plasma insulin

**Figure 5:**
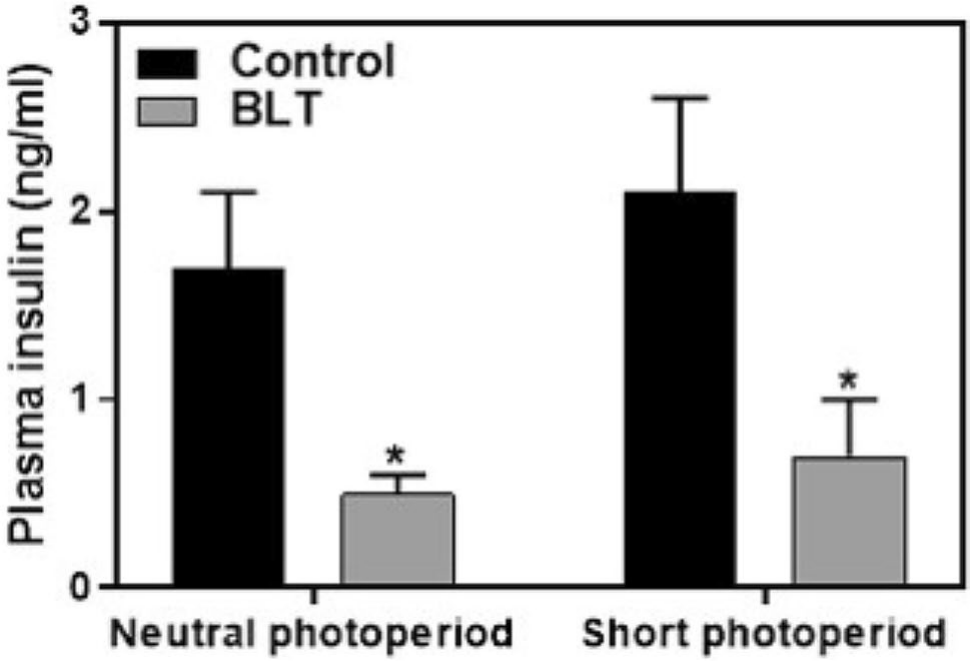
Plasma insulin levels (Means + SEM): Sand rats treated with bright light had significantly lower insulin levels compared with controls in both photoperiods (*p < 0.001).

Data did not follow normal distribution and were analyzed using non-parametric Kruskal–Wallis test. Photoperiod length had no effect on plasma insulin levels [H(1,37) = 0.78, *p* = 0.38]. However, bright light exposure had a strong and significant effect, reducing insulin levels [Fig. 5;H(1,37) = 12.0, *p* = 0.0008; Cohen’s d = 1.35]. Additional data are presented in the supplementary information (Table A4).

### Activity rhythm and plasma insulin

**Figure 6:**
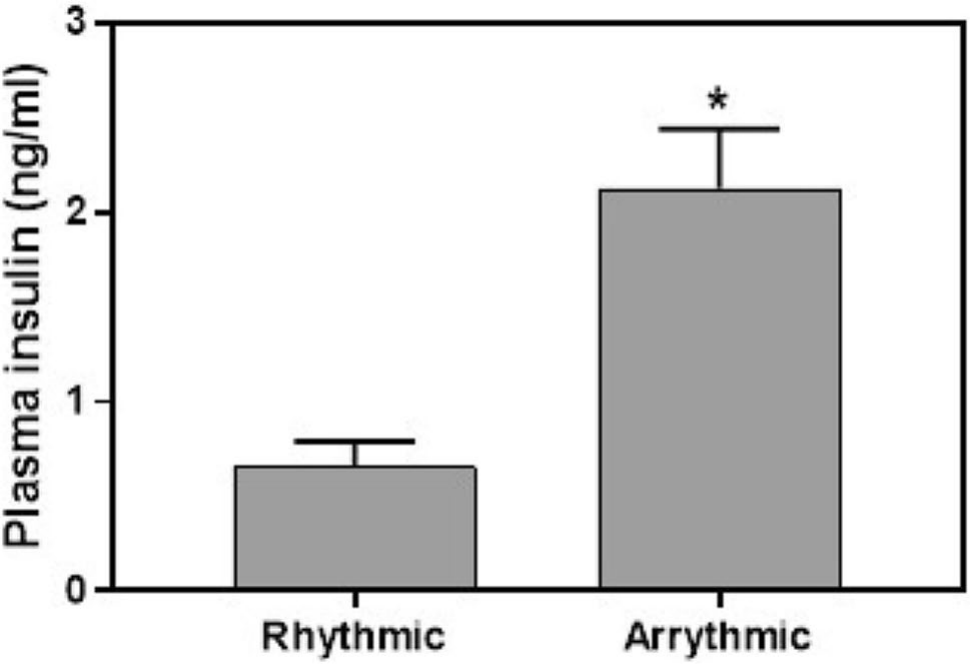
Plasma insulin levels (ng/ml, Means + SEM) of rhythmic compared with arrhythmic sand rats. *p < 0.0001.

Data did not follow normal distribution and were analyzed using non-parametric Kruskal–Wallis test. Plasma insulin in rhythmic animals was significantly lower than in the arrhythmic (see methods) sand rats (Fig. 6; H(1,32) = 13.7, *p* = 0.0002; Cohen’s d = 1.71). This difference was maintained when analysis was performed separately for each group (data not shown). Additional data are presented in the supplementary information (Table A5).

### Cataract

Photoperiod conditions had no effect on the development of mature cataract [χ^2^(1) = 0.03, *p* = 0.86]. However, BLT had a near significant effect, reducing the development of mature cataracts (Control-NP: 5/33, BLT-NP: 1/29, Control-SP:4/25, BLT-SP: 2/30) [χ^2^(1) = 2.91, *p* = 0.09].

### Heart weight

**Figure 7:**
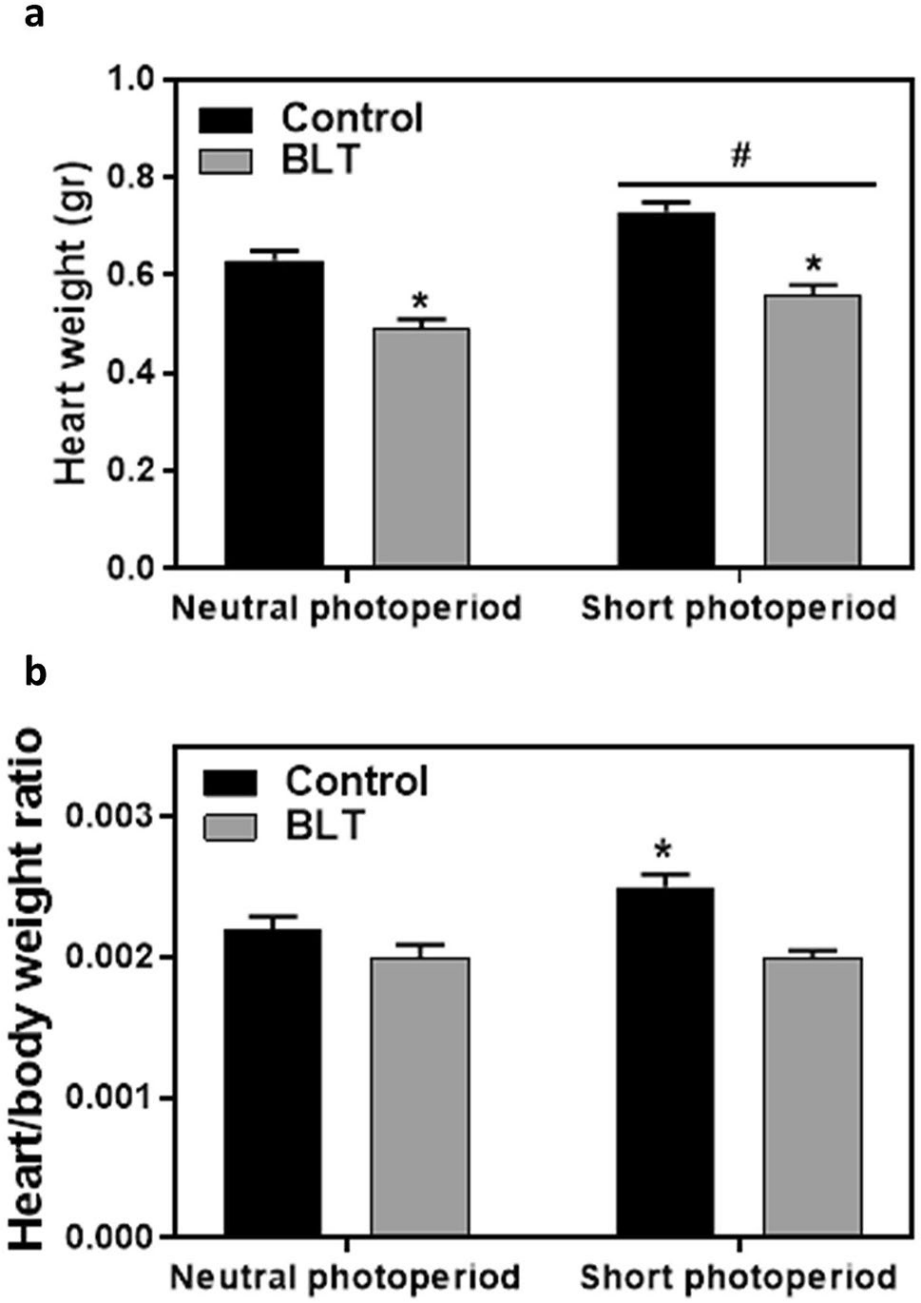
Heart weight (Mean + SEM): Short photoperiod conditions resulted in significantly increased heart weight compared with neutral photoperiod conditions (^#^*p* < 0.001) (**a**), whereas BLT significantly reduced heart weight (**a**) and heart/body weight ratio (**b**) compared with controls (*p < 0.001).

Short photoperiod conditions resulted in significantly increased heart weight [F(1,109) = 13.7, *p* < 0.001; Cohen’s d = 0.5] and borderline significant heart/body weight ratio [F(1,109) = 3.7, *p* = 0.058] (Fig. 7a). Moreover, BLT had a significant effect, reducing heart weight [F(1,109) = 48.4, *p* < 0.001; Cohen’s d = 1.2] (Fig. 7a) and heart/body weight ratio [F(1,109) = 28.6, *p* < 0.001; Cohen’s d = 1.1] (Fig. 7b). No significant interactions between photoperiod conditions and BLT effects were shown. Additional data are presented in the supplementary information (Tables A6 and A7).

### Body weight

**Figure 8:**
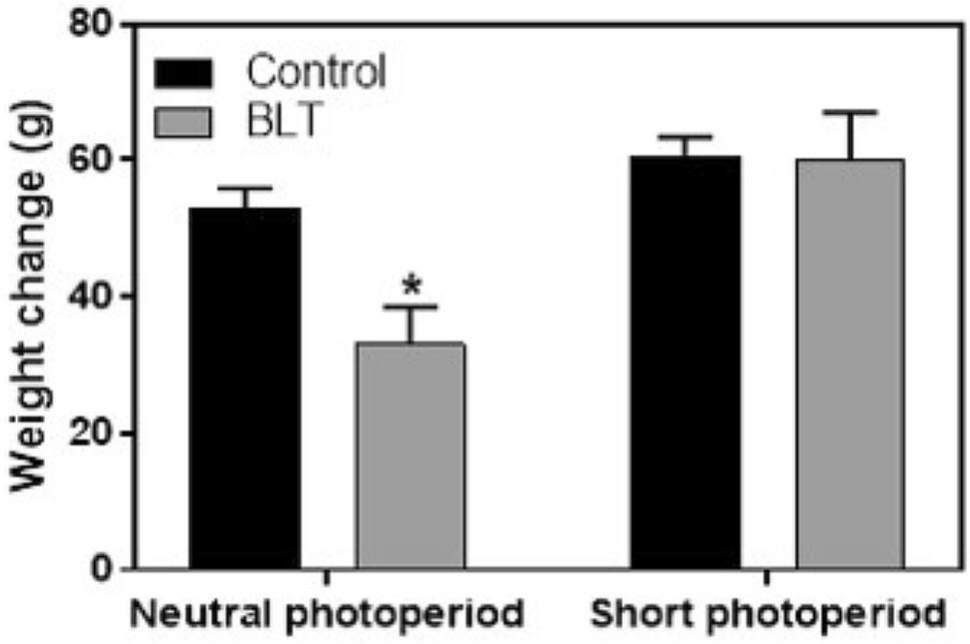
Weight changes throughout the entire experiment (Means + SEM). Animals from the NP-BLT group showed reduced weight gain compared with all other groups (*p = 0.01).

No overall significant differences in weight were identified between neutral and short photoperiod animals [F(1,115) = 0.6, *p* = 0.45] or between animals that were exposed or not exposed to BLT [F(1,115) = 1,0, *p* = 0.32]. However, a significant effect of weight change over time was clearly demonstrated for both photoperiod [F(1,114) = 13.28, *p* < 0.001; Cohen’s d = 0.6] and BLT exposure [(F(1,114) = 4.38, *p* = 0.038; Cohen’s d = 0.3] as well as photoperiod X BLT interaction [F(1,114) = 4.05, *p* = 0.046]. Post-hoc analysis demonstrates that these differences are related to only one group, BLT-NP, that shows lower weight increase compared with other groups (Fig. 8, post-hoc LSD–BLT-NP ≠ all other groups, *p* < 0.01). Additional data are presented in the supplementary information (Table A8).

### Behavioral tests

**Figure 9:**
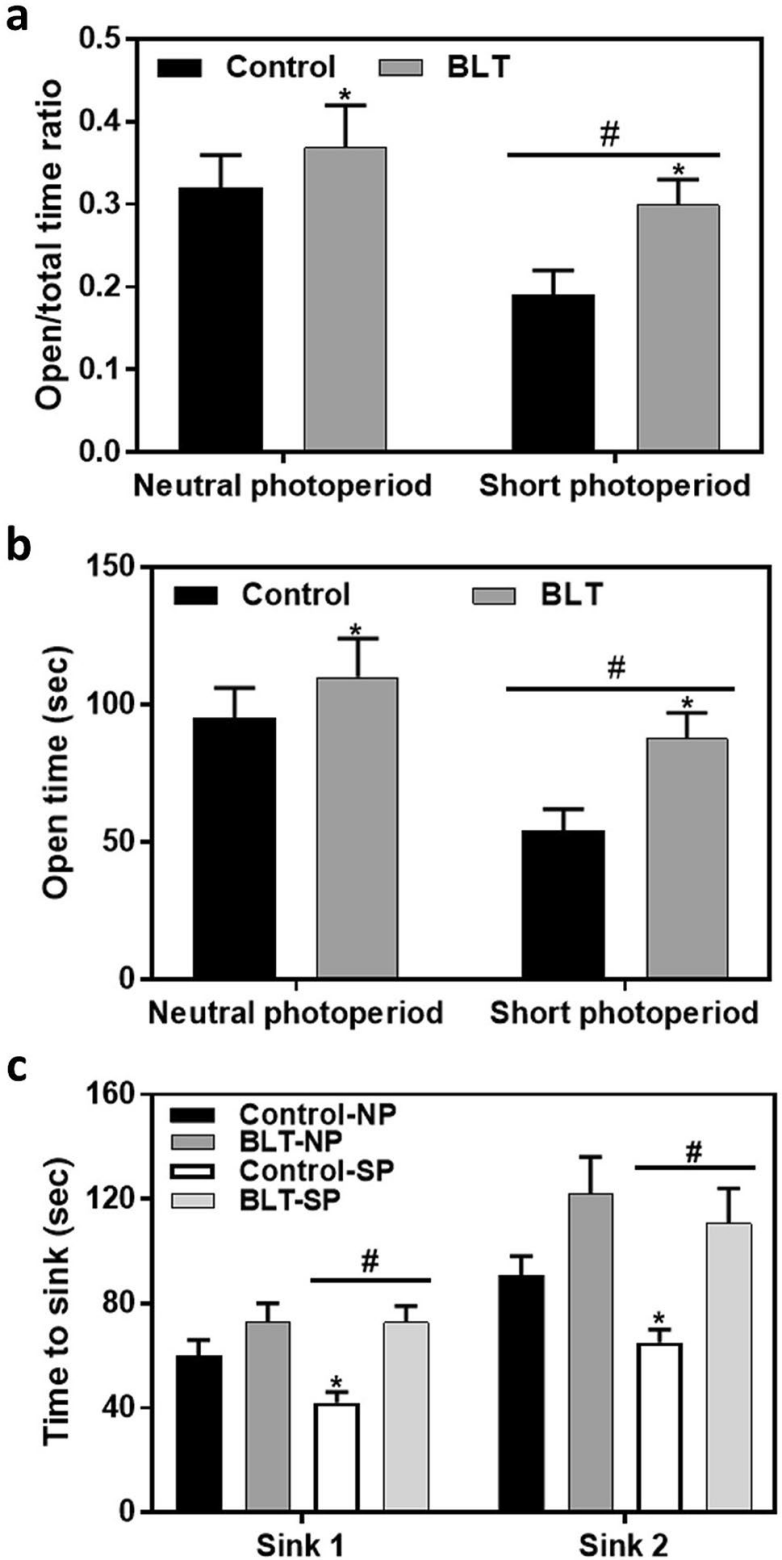
Behavioral tests: Short photoperiod conditions resulted in a significantly shorter time spent in the open arms of the maze compared with neutral photoperiod acclimated sand rats, showing lower open/total time ratio measure (**a**) (^#^*p* = 0.008), as well as reduced time in the open arms measure (**b**) (^#^*p* = 0.034). Bright light treatment increased both the open/total time ratio measure (**a**) (*p = 0.005), and the open time measure (**b**) (*p = 0.028). Animals maintained under short photoperiod demonstrated faster time to sink in the modified FST although statistical significance was only at borderline levels (**c**) (^#^*p* = 0.053). Bright light treatment resulted in significantly longer time to sink in the modified FST (*p < 0.001) (**c**). Bars represent Means + SEM.

Measuring anxiety like behavior in the Elevated plus maze (EPM) we found that animals maintained in short photoperiod conditions spent less time in the open arms of the EPM as demonstrated by lower open/total time ratio measure [Fig. 9a; F(1,60) = 7.55, *p* = 0.008; Cohen’s d = 0.7] as well as reduced time in the open arms measure [Fig. 9b; F(1,60) = 8.5, *p* = 0.005; Cohen’s d = 0.7]. Bright light treatment resulted in an opposite effect in both measures. For ratio measure: Fig. 9a; [F(1,60) = 4.71, *p* = 0.034; Cohen’s d = 0.5]; for open time measure: Fig. 9b, [F(1,60) = 5.01, *p* = 0.028; Cohen’s d = 0.5]. No interaction was shown between photoperiods and BLT in any of the measures [for open time: F(1,60) = 0.85, *p* = 0.36; for open/total time ratio: F(1,60) = 0.95, p = 0.33]. Additional data are presented in the supplementary information (Tables A9 and A10).

Animals maintained in short photoperiods demonstrated faster time to sink in the modified FST although statistical significance was only at borderline levels [Fig. 9c; F(1,58) = 3.89, *p* = 0.053; Cohen’s d = 0.4]. Furthermore, the administration of bright light resulted in significantly longer time to sink in the modified FST [Fig. 9c; F(1,58) = 19.35, *p* < 0.001; Cohen’s d = 0.9]. No significant interaction was shown between photoperiod length and bright light administration [F(1,58) = 1.37, *p* = 0.25] suggesting that the effects of bright light in the FST are not different for animals maintained in neutral or short photoperiods. Additional data are presented in the supplementary information (Table A11).

### PER2 mRNA expression levels

#### SCN

**Figure 10:**
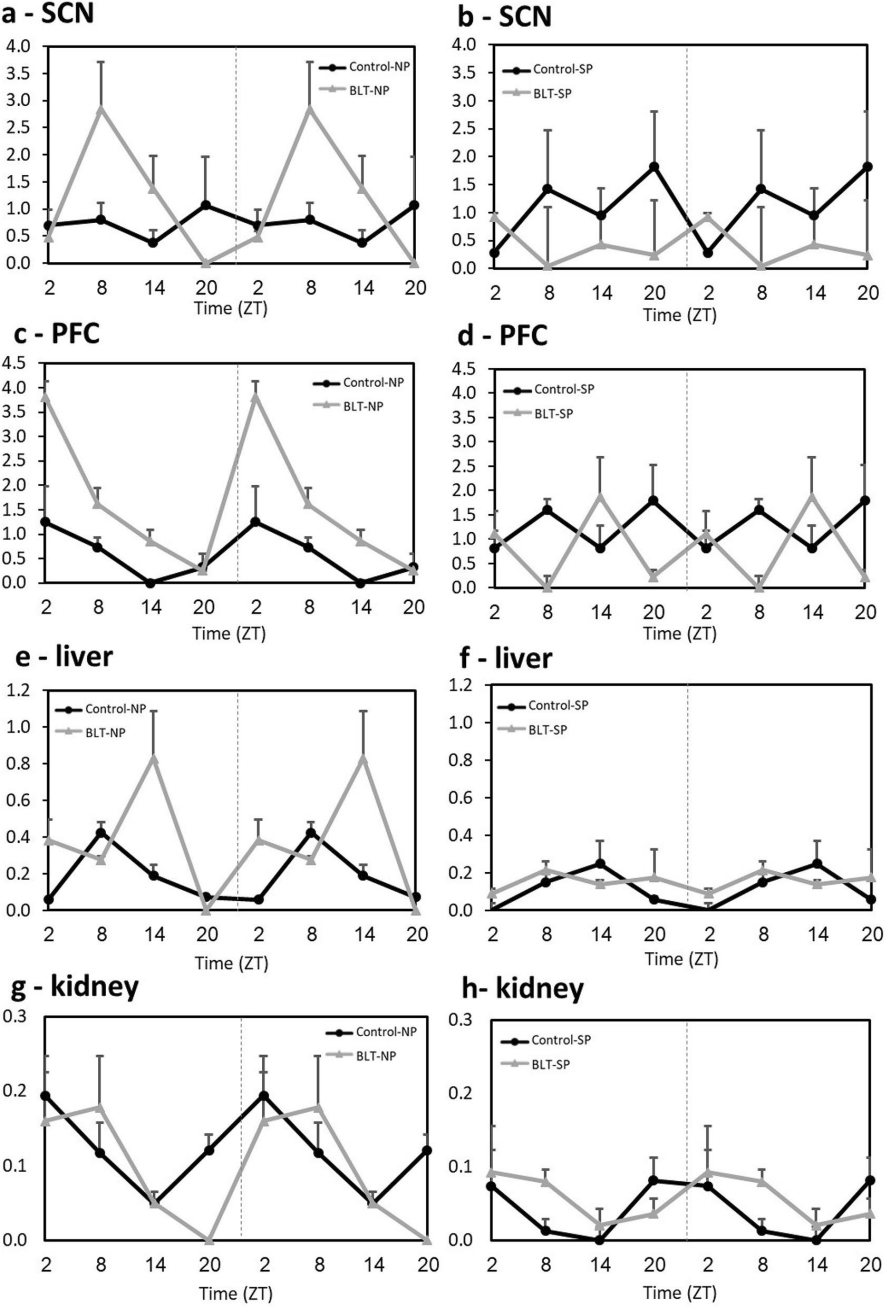
Daily rhythm of mRNA levels of Per2 in the SCN (**a**,**b**), PFC (**c**,**d**), liver (**e**,**f**) and kidney (**g**,**h**) of sand rats kept under neutral (**a**,**c**,**e**,**g**) or short (**b**,**d**,**f**,**h**) photoperiods with or without BLT. Transcript levels were measured by qRT-PCR and normalized to β-actin. Results are the mean + SEM (n = 12–2 for each data point). Broken lines separate double-plotted data. See Table 2 for statistical analyses.

**Figure 11:**
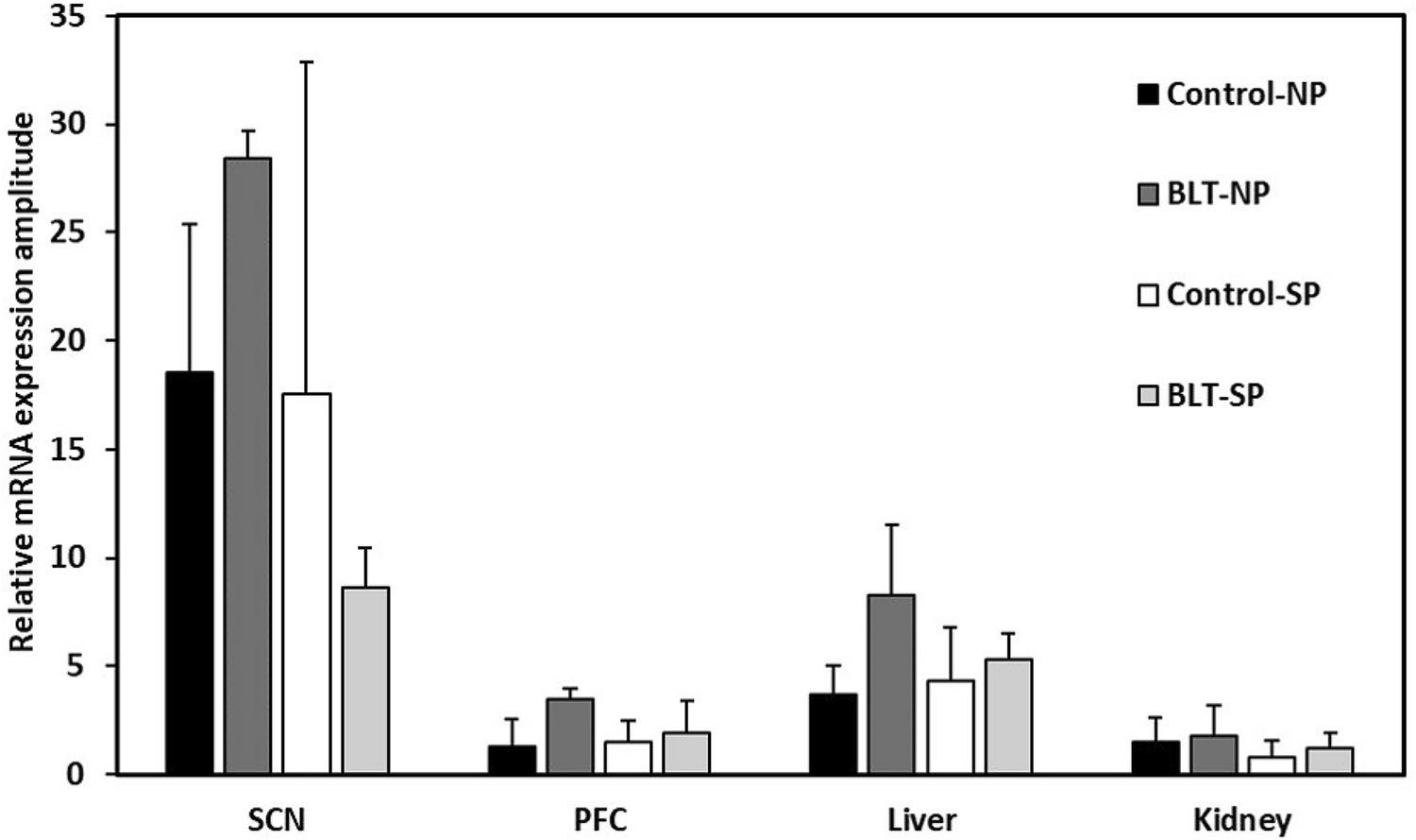
PTDs of Per2 mRNA levels of in the SCN, PFC, liver and kidney of sand rats kept under neutral or short photoperiods with or without BLT. Transcript levels were measured by qRT-PCR and normalized to β-actin. Results are the mean + SEM. See Table 3 for statistical analyses.

ANOVA analysis indicates a rhythm in the BLT groups but not in the control groups, regardless of photoperiod conditions (Fig. 10a,b, see Table 2 for statistical analyses results). Comparison of the peak-trough difference (PTD) clearly demonstrates that the PTD of the neutral photoperiod-BLT group is significantly larger than the PTD in any of the other groups (Fig. 11). No additional significant differences were shown between the other groups (see Table 3 for statistical analyses results). Additional data are presented in the supplementary information (Table A12).

**Table 2:** Per2 mRNA levels ANOVA results across ZT for each group (significant results in bold).

	Control-NP	BLT-NP	Control-SP	BLT-SP
SCN	F(3,17) = 0.2, *p* = 0.9	**F(3,10) = 4.8, p = 0.025**	F(3,15) = 0.6, *p* = 0.65	**F(3,9) = 17.9, p = 0.0004**
PFC	F(3,14) = 1.0, *p* = 0.42	**F(3,9) = 9.45, p = 0.004**	F(3,13) = 0.65, *p* = 0.6	F(3,14) = 1.5, *p* = 0.25
Liver	**F(3,29) = 17.7, p < 0.0001**	**F(3,22) = 6.1, p = 0.004**	F(3,21) = 2.3, *p* = 0.11	F(3,26) = 0.7, *p* = 0.57
Kidney	F(3,27) = 1.6, *p* = 0.22	F(3,23) = 1.5, *p* = 0.24	F(3,20) = 0.5, *p* = 0.68	F(3,25) = 0.9, *p* = 0.46

**Table 3:** Comparison of the peak to trough difference of Per2 mRNA levels across groups (t-test, significant results in bold).

	Control-NP versus Control-SP	BLT-NP versus BLT-SP	Control-NP versus BLT-NP	Control-SP versus BLT-SP
SCN	t(17) = 1.0, *p* = 0.32	**t(12) = 22.8, p < 0.0001**	**t(15) = 3.0, p = 0.008**	t(14) = 1.0, *p* = 0.33
PFC	t(15) = 0.4, *p* = 0.72	**t(11) = 2.27, p = 0.044**	**t(11) = 3.65, p = 0.004**	t(15) = 1.24, *p* = 0.24
Liver	t(26) = 0.98, *p* = 0.34	**t(26) = 3.85, p = 0.0007**	**t(23) = 5.0, p < 0.0001**	t(29) = 0.73, *p* = 0.47
Kidney	t(21) = 1.76, *p* = 0.09	**t(27) = 2.25, p = 0.03**	t(21) = 0.57, *p* = 0.57	t(27) = 0.25, *p* = 0.8

#### PFC

ANOVA analysis indicates a rhythm only in the Neutral photoperiod-BLT group (Fig. 10c,d, see Table 2 for statistical analyses results). Comparison of the PTDs clearly demonstrates that the PTD of the neutral photoperiod-BLT group is significantly larger than that of any of the other groups (Fig. 11). No additional significant differences were shown between the other groups (see Table 3 for statistical analyses results). Additional data are presented in the supplementary information (Table A13).

#### Liver

ANOVA analysis indicates a rhythm in the two groups maintained in the neutral photoperiod conditions and no rhythm in the groups maintained in short photoperiods conditions, regardless of BLT (Fig. 10e,f, see Table 2 for statistical analyses results). Comparison of the amplitudes clearly demonstrates that the PTD of the neutral photoperiod-BLT group is significantly larger than the PTD of any of the other groups (Fig. 11). No additional significant differences were shown between the other groups (see Table 3 for statistical analyses results). Additional data are presented in the supplementary information (Table A14).

#### Kidney

ANOVA analysis within groups did not indicate a significant rhythm (values of at least one time point were significant than the others) in any of the groups, even though a clear trent can be seen in the figure (Fig. 10g,h, see Table 2 for statistical analyses results). However, analysis of the PTD shows that the PTD in animals that were maintained in neutral photoperiods and received BLT was significantly higher compared with animals that were maintained in short photoperiod and received BLT. Moreover, there was a trend for a larger PTD in animals maintained in neutral photoperiod without BLT compared with animals maintained in short photoperiods without BLT. Together it can be suggested that the rhythm in animals maintained in neutral photoperiods was more pronounced compared with animals maintained in short photoperiods (Fig. 11, see Table 3 for statistical analyses results). Additional data are presented in the supplementary information (Table A15).

## Discussion

We found that exposing diurnal sand rats to 1 h of BLT in the morning has a significant, beneficial effect on daily rhythms, blood glucose levels, glucose tolerance, body mass and depressive like behavior, and a near significant effect on the development of cataract. Effect sizes (Cohen’s d) for most of these differences were between medium and large. These results confirm our hypothesis that the beneficial effects of keeping sand rats outdoors, which we reported in a previous study^33^ result from their exposure to high intensity light. Sand rats are diurnal in nature^41^, but as we found in previous studies^17,36^, when kept indoors under standard laboratory conditions they are nocturnal or arrhythmic: general activity of 42% of the control individuals (both photoperiods combined) was significantly nocturnal, and the rest 58% were arrhythmic. Moreover, under both photoperiod conditions no significant daily rhythms were found in blood glucose levels and in per2 gene expression in the SCN, PFC, and kidney.

Comparing animals kept under short and neutral photoperiod we found that in accordance with our previous studies^17,19,32,33,39,40,42–45^, there was a higher number of arrhythmic individuals in the short photoperiod group. They also had lower glucose tolerance, higher fasting blood glucose levels, higher heart weight, higher increase in body mass, and higher anxiety- and depression-like behavior. There were no differences in the daily peak to trough difference (PTD) of per2 expression levels in the SCN, PFC, liver and kidney between animals maintained in the different photoperiod conditions. In the liver, a significant rhythm in per2 expression was found in animals kept under neutral, but not under short photoperiod.

We have previously found that when kept outdoors, where they are exposed to numerus biotic and a-biotic cycling variables and to un-controlled conditions, sand rats are diurnal and do not develop circadian rhythm-related pathologies compared to control sand rats kept in controlled laboratory conditions with similar photoperiod^33^. Our main hypothesis in the current study was that BLT in the morning will be sufficient in order to ameliorate the circadian rhythm-related pathologies by resynchronizing disrupted circadian rhythms and increasing rhythms PTD. Indeed, we found that in response to long-term BLT in the morning, most individuals under both photoperiods were rhythmic, demonstrated a significant daily rhythm in blood glucose levels, and in per2 gene expression in the SCN. Interestingly, rhythmic individuals in the BLT-SP group where nocturnal, while rhythmic individuals from the BLT-NP group were diurnal. In the BLT-NP group, light treatment also restored the daily rhythm of per2 gene expression in the PFC. In both NP groups, per2 gene expression in the liver was rhythmic. As we expected, BLT also resulted in an increase in per2 expression PTD, but only in the NP acclimated sand rats; its PTD was significantly larger than the other three groups in all tissues tested except in the kidney, where a significantly larger PTD was found only in the BLT-NP group compared to both SP groups. Bright light treated animals from both photoperiods were also healthier: they were normoglycemic, had lower heart weight, near significant lower incidence of cataract, and showed lower anxiety- and depression-like behaviors compared with sand rats that were not treated with bright light. BLT-NP groups also gained less mass during the experiment. In a previous study we found that photoperiod rather than diet had a significant effect on systolic blood pressure which was elevated in short photoperiod-acclimated animals^33^. This, in addition to the more prevalent glucose intolerance and higher basal glucose levels in the short photoperiod groups, which is a known reason for cardiac hypertrophy^46,47^, could explain the higher heart weight in the SP acclimated groups. Information about ambient light conditions is relayed through the eyes ipRGCs to different brain areas including the SCN, the lateral geniculate nucleus, the subparaventricular zone of the hypothalamus, medial amygdala, and the lateral habenula^48^, areas involved in diverse functions. Therefore, it is not surprising that light treatment has such a wide range of biological effects beyond vision.

Here we show that BLT restores circadian rhythmicity in sand rats, and ameliorates the metabolic and affective effects of laboratory conditions and short photoperiod exposure. We conclude that dampened behavioral, physiological and molecular daily rhythms of sand rats under these conditions increase their susceptibility to T2DM and depression-like behavior. We suggest that the disturbed rhythms disrupt the internal temporal order, resulting in the development of these disorders. Furthermore, we suggest that BLT may prevent, or at least delay the development of T2DM and depressive-like behavior in the sand rats by entraining circadian rhythms and increasing their amplitude.

Light has a complex, time-dependent effect on animal and human health^49^. Studies in both animals and humans suggest that exposure to light at night, or light pollution, has negative effects on health^50–54^, while exposure to light during the day promotes health^9,29,49,55–61^. Studying the effects of light exposure in humans, and especially the effects of daylight, is limited technically and most reported studies are observational or correlational^49^. At the same time, animal studies are almost always conducted on mice and rats, which are active during the night (dark) and rest in their dark borrows during the day, hence hardly ever exposed to light. Nocturnal and diurnal mammals have opposite activity patterns, however the function of their circadian system and its molecular mechanisms are very similar, both in mechanisms and phase^31^. Moreover, light is an aversive, anxiogenic environmental signal for nocturnal, but not diurnal species^31,32,39,40,62^. Therefore, we suggest that the common practice of using nocturnal models can restrict interpretations to human research and health significantly, especially when studying the importance of daylight exposure for human health and well-being, or circadian rhythm-related diseases like depression and T2DM, and that the sand rat is an excellent animal model for such studies.

Finally, our findings may have very important implications for the management and prevention of cardiometabolic and mental health disorders that are very prevalent in humans. In many ways, laboratory conditions are similar to the modern Western lifestyle and living conditions: they are characterized by controlled ambient temperature and constant food availability^63^, sedentary way-of-life, no interspecific interactions, extensive use of artificial light during the night ("light pollution") and low exposure to daylight^18^. Consequently, it has been suggested that the resulting circadian rhythm disturbances may be a major contributor to the contemporary global epidemics of T2DM, CVD and obesity through what we have recently termed the "Circadian Syndrome"^18^. Therefore, a better understanding of the relationship between modern lifestyle, circadian disruption, cardio-metabolic disorders such as heart disease, T2DM, and depression, may lead to improved health and well-being as well as circadian medicine approaches based on non-pharmacological prevention and therapeutic strategies, like BLT and controlled light exposure.

## Methods

### Animals

The study involved 131 HsdHu diabetes-prone 10–11 months old male fat sand rats (*Psammomys obesus*) from our colony at the Zoological Research Garden, Tel Aviv University. As previously described^33^, animals were individually housed in standard plastic cages (42 cm, 26 cm, 15 cm), containing only bedding, food and water, positioned in temperature-controlled rooms (25 °C), and provided *ad-lib* tap water and standard rodent food (product 19510; Koffolk, Petach-Tikva, Israel).

### Procedure

Before the experiments started, all animals were maintained on a low-energy diet (product 1078, Koffolk Ltd, Israel) to prevent the development of diabetes. All animals were weighed and tested for glucose tolerance before the start of the experiment. The experiment was structured in a 2 × 2 design, with day length (SP, 5:19; NP, 12:12 LD, 800 lux, wavelength 420–780 nm, 5834 K, Figure A1a) and bright light treatment (3,000 lux, wavelength 420–780 nm, 5487 K, Figure A1b, for 1 h administered at ZT 0–1) as main independent variables. The short day lighting regime was designed according to the sand rats' winter/summer activity in nature, where during winter they are active outside their burrows for about 5 h daily around mid-day, while during summer their activity starts in the early hours of the morning and ends in the late afternoon (about 12 h from activity onset to offset), with a pause during the mid-day hours of extreme desert heat^64,65^, and to previous results, showing that 3 weeks exposure to such photoperiod results in a depression-like behavioral change^42^. Eight hundred lux is the standard laboratory light illuminance, and is also common light intensity in a well-lit office. These conditions simulate the insufficient exposure to high-intensity light during the day in modern western lifestyle.

Animals were assigned to the experimental groups based on weight and blood glucose levels to avoid a baseline bias, n = 31–34/group in a room with the following conditions: short photoperiod, no BLT (Control-SP), neutral photoperiod, no BLT (Control-NP), neutral photoperiod with BLT (BLT-NP) and short photoperiod with BLT (BLT-SP). Body weight was measured weekly during the experiment. In-cage locomotor activity was monitored throughout the experiments in 12 animals in each treatment group using IR motion detectors (Orev Ltd., Israel), except from the BLT-SP group, where only 6 individuals were monitored due to a technical problem. On week 16, around ZT 2, animals were weighed, blood (from the tail tip) was collected for glucose levels was measurement (U-Right glucometer TD -4269, TaiDoc, New Taipei City, Taiwan), plasma was frozen for insulin measurement and oral glucose tolerance tests were performed. The plasma samples of 10 animals from each treatment were assayed for insulin. Behavioral tests for anxiety- and depressive-like behavior were conducted on week 17. On week 18 the animals’ eyes were examined for the presence of cataracts (a common complication of T2DM). After the end of the above mentioned experiments, sand rats were euthanized at 4 different time points (at 6-h intervals; ZT2, ZT8, ZT14, ZT20 (ZT = zeitgaber time, ZT 0 = lights on); n = 6–12 for each time point in each group), and we collected blood, SCN, PFC, kidney, and liver. All tissues were snap-frozen for the analysis of Per2 expression levels using RT-PCR. The chest cavity was rapidly opened, the heart removed and weighed, and the heart weight/body weight ratio calculated.

All experimental procedures followed the NIH guidelines for the care and use of laboratory animals and were approved by the Institutional Animal Care and Use Committee (IACUC) of Tel Aviv University (permit number L15055).

### Photoperiod conditions and bright light treatment

Sand rats were acclimated to short photoperiod (SP, 5:19 LD, n = 66) with lights on at 09:00 and off at 14:00 or to neutral photoperiod (NP, 12:12 LD, n = 65) with lights on at 09:00 and off at 21:00. Light sources were LED tapes located about 40 cm above the cages. Light spectrum and intensity of regular lighting and BLT were recorded using Sekonic Spectromaster C-700 (North White Plains, NY, USA). Light during the light period was 800 lux at wavelength 420–780 nm, 5834 K (Fig. A1).

**Figure 12:**
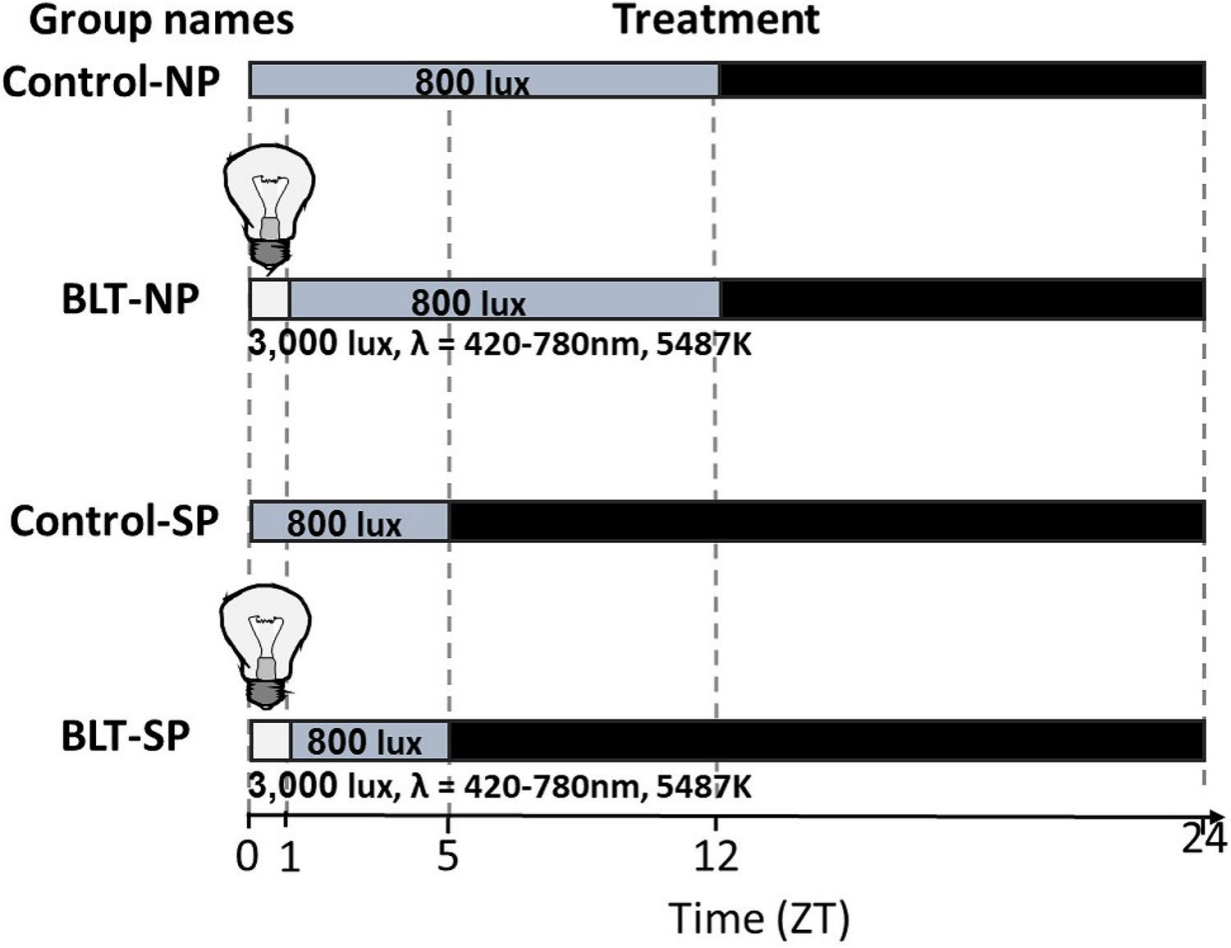
Experimental design. Control-NP = Neutral photoperiod, BLT-NP = Neutral photoperiod with bright light treatment, Control-SP = Short photoperiod, BLT-SP = Short photoperiod with bright light treatment. λ = wavelength.

Each photoperiod group was further divided into two subgroups (n = 31–34/subgroup): “Control” (n = 33–34) and “Bright light treatment” (BLT, n = 31–33) (wide spectrum LED light, 3000 lux, wavelength 420–780 nm, 5487 K, Fig. A1). Light treatment was administered daily during the first hour from “lights on” (ZT0-ZT1). Light treatment was administered for 16 weeks prior to the starting of testing and continued during the days of testing (Fig. 12). The animals had no access to a shelter, and therefor were all exposed to BLT equally.

### Oral glucose tolerance test (OGTT)

Glucose tolerance tests were performed on week 16 in animals fasted for 4 h. Based on previous results^17,19,33^ and to avoid un-necessary stress, blood was collected from the tip of the tail at time 0 and 120 min only. Tests were performed at ZT 2 by administering 2 g glucose/kg body weight dissolved in 1 ml water using gastric gavages (a syringe attached to a 20-gauge, 1.5 feeding needle), inserted through the mouth into the stomach.

### Behavioral tests

On week 17, animals were evaluated in two standard behavioral tests for depression- and anxiety-like behaviors: first the Elevated Plus-Maze (EPM) and 4 days later the modified Forced Swim Test (FST).

*Elevated plus-maze (EPM)* This test presents the rodent with a conflict between its tendency to remain in a safe enclosed dark area and the need to explore new environments^66,67^. For the present study, we followed our standard procedure^30,39,43,68^. The maze was constructed from black aluminum and had two open arms (50 cm long and 10 cm wide) and two closed arms (same dimensions with 15 cm high walls). The plus maze was elevated 50 cm above the floor and light levels at the open arms were 200 lux. The test started an hour after light onset in the experimental rooms (09:00) and animals were tested only during the next 2 h. Order of testing was random within group. Sand rats were individually placed in the center of the maze and their behavior digitally recorded from above for a 5 min session. Recordings were used for later manual scoring of behaviors. At the end of the session, animals were returned to their cages and the maze was wiped clean with 70% ethanol before the start of the next session. Scoring included the time and the number of entries into each arm and was done by an investigator blind to treatment.

*Forced swim test* is a commonly used screening test for the evaluation of depression-like behavior and assessment of antidepressants effects^69^. As described in our previous papers, the FST was used with several methodological alterations^17,32,33,39,40,42–45,70^. Each animal was subjected to the FST twice over two consecutive days with the second exposure serving as the test session. Testing started an at least an hour after the onset of lights and ended within the light period in the experimental rooms. Each animal was placed individually into a white opaque cylinder, 30 cm in diameter and 45 cm high, filled with water (22–23 °C) to a depth of 25 cm. The test was digitally recorded from above for later manual scoring of behavior. As noted in previous work with sand rats, their ability to float is lower compared with rats or mice. Therefore, the standard measure of floating (or immobility) time in the FST was replaced with the measure of “time to sink” where a sink event is defined by the animal going entirely under water for approximately 2 s. Accordingly, right after the second sink, animals were taken out of the water by the experimenter and placed in their home cage and the test was terminated. Water in the cylinder was replaced after each test. Recordings were used to score the time of sink events by an experimenter blind to treatments.

### Activity patterns

The significance of the circadian rhythm in locomotor activity was calculated by χ^2^ test using CTools 7.0 software by van der Veen. In case no significant rhythm was found, the individual was considered arrhythmic. In the rhythmic individuals, an individual was defined as diurnal if more than 50% of its activity occurred during the light phase, and nocturnal if more than 50% of its activity occurred during the dark phase^36,71,72^.

### Heart weight

As previously described^17,33^, on week 18, the sand rats were euthanized, the chest cavity was rapidly opened, the heart removed and rinsed in two washes of ice-cold saline. Major blood vessels and connective tissue were removed, the heart blotted dry, weighed, and the heart weight/body weight ratio calculated.

### Plasma insulin ELISA

Blood samples of 10 animals from each treatment were taken at ZT2 and assayed for insulin. Plasma insulin protein was assayed with an immunoassay ELISA kit (Rat Insulin Ultrasensitive, ALPCO, Salem, NH).

### RNA extraction and RT-PCR

Total RNA was isolated from SCN, PFC, liver, and kidney tissue using the QIAGEN RNeasy Mini Kit (Qiagen, CA, USA) according to the manufacturer’s instructions. RNA levels were determined by the absorbance at 260 nm. The relative expression of Actin and Per2-mRNA was assessed by RT-PCR using equal levels of cDNA (0.5 mg for the brain sample). Reverse transcription (42 °C for 30 min) and PCR (35 cycles consisting of denaturation at 95 °C for 1 min, annealing at 64 °C for 1 min and synthesis at 72 °C for 1 min) were performed for Per2 and Actin. The following primer sequences were used: Per2: f-tcactcaggagtgcatggag, r-tggtgtttcccaacactgac; Actin: f-atgcctgggtacatggtggt, r-cacggacctctacgccaa. Primers were purchased from Sigma-Aldrich (Israel). Comparison between 2 photoperiod groups was performed using the same levels of total RNA at all ZT points, and with the same sampling procedures. Results were normalized to Actin^33^.

#### Cataracts

Local anesthetics was achieved with local administration of Oxybuprocaine hydrochloride 0.4% (Localin, Fisher Pharmaceuticals Labs, Bnei Brak, Israel). Then the pupils were dilated using topical administration of one drop of 0.5% tropicamide eye drops (Mydramide, Fisher Pharmaceuticals Labs, Bnei Brak, Israel) and one drop of Phenylepheine hydrochlorie 10% eye drops (Erin 10% 5 ml Fisher Pharmaceuticals Labs, Bnei Brak, Israel). Slit lamp examination (Keeler PSL classic portable slit lamp) was performed to evaluate the lens. The ophthalmic examination was performed in masked fashion (Ophthalmologist was unaware of the treatment methods of the examined animal). All finding were documented in a coded spread sheet caring only the animal code. Cataract was defined as lens opacity in accordance with ICD-9 code 366.9.

#### Data analysis

Statistical analysis was performed using Statistica 13.2 (Dell, Tulsa, OK). Two-way ANOVA was used to analyze data from all experiments followed by LSD post-hoc tests when needed. Photoperiod and treatment were the main factors (short or neutral for photoperiod and BLT or control for treatment).When data were not normally distributed, Kruskal–Wallis non-parametric tests were used for analysis. We computed effect sizes for Kruskal–Wallis tests as the eta squared based on the H-statistic: eta2[H] = (H—k + 1)/(n—k); where H is the value obtained in the Kruskal–Wallis test; k is the number of groups; n is the total number of observations as suggested by Tomczak & Tomczak^73^. We used an appropriate online calculator for these computations (https://www.psychometrica.de/effect_size.html).

The actograms and the significance of the circadian rhythm in locomotor activity was calculated by χ^2^ test using CTools 7.0 software by van der Veen. Activity pattern was defined as diurnal if more than 50% of activity occurred during the light phase, and nocturnal if more than 50% of activity occurred during the dark phase. To identify the existence of circadian rhythms in glucose levels as well as in per2 mRNA expression levels we used one-way ANOVAs with ZT as main factor within each group to explore differences between levels at different ZT points. Significant ANOVA results were followed by LSD post-hoc test. A rhythm was considered significant when there was a significant difference between at least two ZT time points^33^. Comparison of PTDs between groups was done using t-tests after computing the largest difference between ZTs in each group and the variance for this difference.

## Supplementary information

Supplementary information.
