# Supplementary material for: Beneficial effects of daytime high-intensity light exposure on daily rhythms, metabolic state and affect
Source: Sci Rep. 2020 Nov 13;10:19782. doi: 10.1038/s41598-020-76636-8 (PMC7666121; doi:10.1038/s41598-020-76636-8)
Supplement: Supplementary file 1 — Supplementary information. [file 41598_2020_76636_MOESM_ESM.pdf]

Beneficial effects of daytime high-intensity light exposure on daily rhythms, metabolic state and affect

Short title: Effects of daytime light exposure on daily rhythms, metabolic state and affect

Carmel Bilu[1], Haim Einat[2], Paul Zimmet[3], Vicktoria Vishnevskia-Dai[4], and Noga Kronfeld-Schor#[1]

[1]School of Zoology, Tel-Aviv University, Tel Aviv, Ramat Aviv, Israel, [2]School of Behavioral Sciences, Tel Aviv-Yaffo Academic College, Tel-Aviv, Israel. [3]Department of Medicine, Monash University, Melbourne, Victoria, Australia, [4]Ocular Oncology and Autoimmune service, The Goldschleger Eye Institute, The Chaim Sheba Medical Center, Tel-Hashomer, Sackler Faculty of Medicine, Tel-Aviv University, Israel.

# Corresponding author: nogaks@tauex.tau.ac.il

Appendix

Light spectra

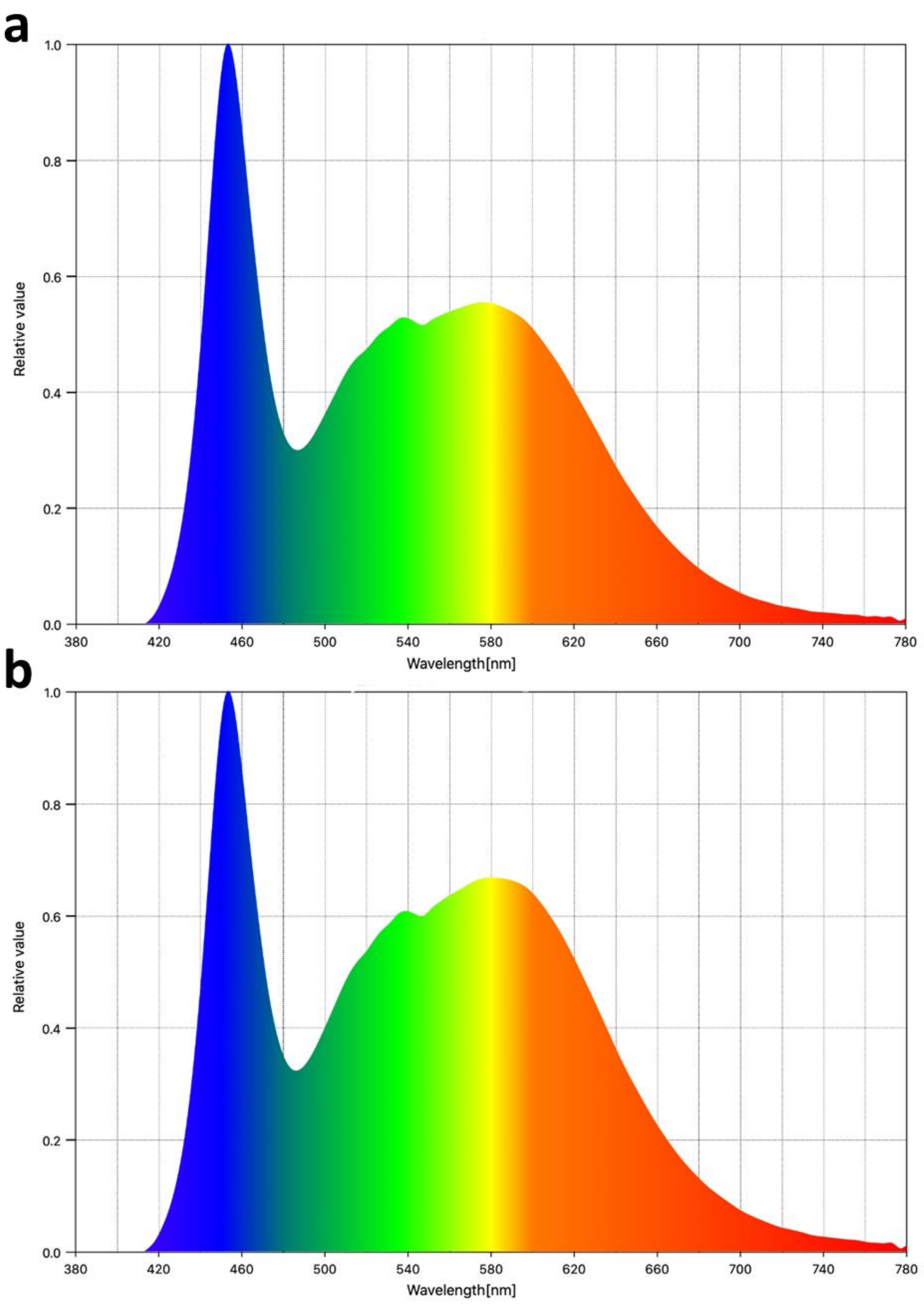

Figure A1. Light spectra of the regular lighting in the rooms: 800 lux at wavelength 400–780 nm (5834 K) **(a)** and BLT: 3,000 lux, wavelength 400–780 nm, 5487 K **(b)**.

Summary of data for study variables

Table A1. Fasting blood glucose levels (mg/dL) at baseline (time 0) and 120-min post-glucose administration (time 120) in the oral glucose tolerance test. Numbers represent mean ± SEM and number of animals in parentheses.

	Control	BLT
Neutral photoperiod - time 0	196±16 (27)	93±10 (27)
Short photoperiod - time 0	230±22 (24)	158±13 (30)
Neutral photoperiod - time 120	235±20 (27)	126±18 (27)
Short photoperiod - time 120	299±24 (24)	196±16 (30)

Table A2. Twenty-four-hour glucose rhythm. Numbers represent mean ± SEM and number of animals in parentheses.

	ZT2	ZT8	ZT14	ZT20
Control-NP	243.5±70.4 (6)	234±35.9 (9)	268±44.9 (6)	198.3±24.5 (12)
BLT-NP	116±28.8 (11)	203.5±13.8 (6)	84.8±11.7 (5)	131.3±34.2 (6)
Control-SP	257±40 (7)	297.3±35.4 (6)	248.8±32 (6)	252.7±64.5 (6)
BLT-SP	162.6±17.7 (12)	233±34 (6)	133.2±29.9 (6)	102.7±26 (6)

Table A3. Blood glucose levels (mg/dl) of rhythmic compared with arrhythmic sand rats. Numbers represent mean ± SEM and number of animals in parentheses.

Rhythmic	105±12 (24)
Arrythmic	378±33 (16)

Table A4. Plasma insulin levels. Numbers represent mean ± SEM and number of animals in parentheses.

	Control	BLT
Neutral photoperiod	1.7±0.4 (9)	0.5±0.1 (11)
Short photoperiod	2.1±0.5 (9)	0.7±0.3 (8)

Table A5. Plasma insulin levels (ng/ml) of rhythmic compared with arrhythmic sand rats. Numbers represent mean ± SEM and number of animals in parentheses.

Rhythmic	0.66±0.13 (19)
Arrythmic	2.13±0.31 (13)

Table A6. Heart weight. Numbers represent mean ± SEM and number of animals in parentheses.

	Control	BLT
Neutral photoperiod	0.63±0.02 (32)	0.49±0.02 (29)
Short photoperiod	0.73±0.02 (24)	0.56±0.02 (28)

Table A7. Heart/body weight ratio. Numbers represent mean ± SEM and number of animals in parentheses.

	Control	BLT
Neutral photoperiod	0.0022±0.00009 (32)	0.002±0.00009 (29)
Short photoperiod	0.0025±0.00009 (24)	0.002±0.00005 (28)

Table A8. Weight changes throughout the experiment. Numbers represent mean ± SEM and number of animals in parentheses.

	Control	BLT
Neutral photoperiod	52.9±3 (34)	33.2±5.3 (29)
Short photoperiod	60.7±2.7 (25)	60.3±6.7 (30)

Table A9. Open/total time ratio in the elevated plus maze. Numbers represent mean ± SEM and number of animals in parentheses.

	Control	BLT
Neutral photoperiod	0.32±0.04 (16)	0.37±0.05 (16)
Short photoperiod	0.19±0.03 (16)	0.3±0.03 (16)

Table A10. Open time in the elevated plus maze. Numbers represent mean ± SEM and number of animals in parentheses.

	Control	BLT
Neutral photoperiod	95±11 (16)	110±14 (16)
Short photoperiod	54±8 (16)	88±9 (16)

Table A11. Time to sink in the forced swim test. Numbers represent mean ± SEM and number of animals in parentheses.

	Sink 1	Sink 2
Control-NP	60±6 (15)	91±7 (15)
BLT-NP	73±7 (16)	122±14 (16)
Control-SP	42±4 (16)	65±5 (16)
BLT-SP	73±6 (15)	111±13 (15)

Table A12. Daily rhythm of mRNA levels of Per2 in the SCN of sand rats kept under neutral or short photoperiods with or without BLT. Numbers represent mean ± SEM and number of animals in parentheses. In the figures data is expressed as the increase from the lowest data point for each tissue.

	ZT2	ZT8	ZT14	ZT20
Control-NP	8.3±3.3 (5)	9.3±3.7 (6)	5.12±2.86 (4)	12.05±9.397 (6)
BLT-NP	6.1±1.7 (3)	29.7±9.3 (3)	15.1±6.62 (4)	1.32±0.52 (4)
Control-SP	4.1±1.2 (4)	15.6±11 (5)	10.78±5.47 (5)	19.55±10.32 (5)
BLT-SP	10.5±1 (4)	1.8±0.89 (3)	5.6±0.55 (3)	3.74±0.67 (3)

Table A13. Daily rhythm of mRNA levels of Per2 in the PFC of sand rats kept under neutral or short photoperiods with or without BLT. Numbers represent mean ± SEM and number of animals in parentheses. In the figures data is expressed as the increase from the lowest data point for each tissue

	ZT2	ZT8	ZT14	ZT20
Control-NP	2.1±0.89 (4)	1.55±0.36 (6)	0.82±0.23 (4)	1.15±0.43 (4)
BLT-NP	4.6±0.48 (2)	2.44±0.49 (4)	1.68±0.4 (4)	1.07±0.17 (3)
Control-SP	1.9±0.53 (5)	2.69±0.4 (4)	1.9±0.63 (4)	2.88±0.91 (4)
BLT-SP	2.2±0.64 (6)	1.1±0.41 (4)	2.96±0.99 (4)	1.32±0.31 (4)

Table A14. Daily rhythm of mRNA levels of Per2 in the liver of sand rats kept under neutral or short photoperiods with or without BLT. Numbers represent mean ± SEM and number of animals in parentheses. In the figures data is expressed as the increase from the lowest data point for each tissue

	ZT2	ZT8	ZT14	ZT20
Control-NP	0.95±0.11 (6)	4.61±0.59 (9)	2.24±0.63 (6)	1.08±0.18 (12)
BLT-NP	4.18±1.15 (10)	3.11±0.24 (6)	8.67±2.62 (4)	0.36±0.04 (6)
Control-SP	0.99±0.42 (7)	2.49±0.64 (6)	3.48±1.26 (6)	1.58±0.21 (6)
BLT-SP	1.88±0.3 (12)	3.14±0.51 (6)	2.38±0.24 (6)	2.76±1.54 (6)

Table A15. Daily rhythm of mRNA levels of Per2 in the kidney of sand rats kept under neutral or short photoperiods with or without BLT. Numbers represent mean ± SEM and number of animals in parentheses. In the figures data is expressed as the increase from the lowest data point for each tissue

	ZT2	ZT8	ZT14	ZT20
Control-NP	2.28±0.61 (6)	1.52±0.48 (9)	0.83±0.13 (6)	1.55±0.29 (10)
BLT-NP	1.94±0.74 (10)	2.12±0.77 (6)	0.84±0.23 (6)	0.34±0.08 (5)
Control-SP	1.46±0.89 (7)	0.85±0.24 (6)	0.72±0.26 (6)	1.53±0.39 (5)
BLT-SP	1.65±0.38 (12)	1.52±0.24 (6)	0.92±0.31 (6)	1.08±0.29 (5)
